# Neuropilin‐2 regulates airway inflammation in a neutrophilic asthma model

**DOI:** 10.1002/iid3.575

**Published:** 2021-12-03

**Authors:** Robert M. Immormino, Corey M. Jania, Stephen L. Tilley, Timothy P. Moran

**Affiliations:** ^1^ Center for Environmental Medicine, Asthma and Lung Biology University of North Carolina Chapel Hill North Carolina USA; ^2^ Department of Medicine University of North Carolina Chapel Hill North Carolina USA; ^3^ Department of Pediatrics University of North Carolina Chapel Hill North Carolina USA

**Keywords:** airway inflammation, alveolar macrophage, efferocytosis, neuropilin‐2, neutrophilic asthma, Toll‐like receptor

## Abstract

**Background:**

Asthma is a heterogenous disease that can be classified into eosinophilic (type 2‐high) and noneosinophilic (type 2‐low) endotypes. The type 2‐low endotype of asthma can be characterized by the presence of neutrophilic airway inflammation that is poorly responsive to corticosteroids. Dysregulated innate immune responses to microbial products including Toll‐like receptor (TLR) ligands have been associated with the pathogenesis of neutrophilic asthma. The key molecules that regulate inflammatory responses in individuals with neutrophilic asthma remain unclear. We previously reported that the immunoregulatory receptor neuropilin‐2 (NRP2) is expressed by murine and human alveolar macrophage (AM) and suppresses lipopolysaccharide (LPS)‐induced neutrophilic airway inflammation.

**Methods:**

Here, we investigated the immunoregulatory role of NRP2 in a mouse model of neutrophilic asthma.

**Results:**

We found that TLR ligands, but not T helper 2 (Th2)‐promoting adjuvants, induced NRP2 expression by AM. Using an LPS‐mediated model of neutrophilic asthma, we demonstrate that NRP2 was increased in AM and other lung antigen‐presenting cells following airway challenge with antigen. Conditional deletion of NRP2 in myeloid cells exacerbated airway inflammation in a neutrophilic asthma model. In contrast, myeloid‐specific ablation of NRP2 did not affect airway inflammation in a Th2‐mediated eosinophilic asthma model. Myeloid‐specific ablation of NRP2 did not affect Th1/Th17 responses to inhaled antigens or expression of neutrophil chemokines but rather resulted in impaired efferocytosis by AM, which is necessary for effective resolution of airway inflammation.

**Conclusion:**

Our findings suggest that NRP2 is a negative regulator of airway inflammation associated with neutrophilic asthma.

## Graphical abstract

Neutrophilic asthma is a subset of asthma that is frequently severe and refractory to standard therapies. The key molecules that regulate airway inflammatory responses in individuals with neutrophilic asthma remain unclear. Here, we show that the protein neuropilin‐2 inhibits airway inflammation in an animal model of neutrophilic asthma.

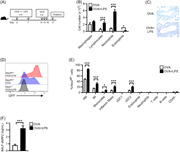

## 1 INTRODUCTION

Asthma is a chronic lung disease characterized by airway inflammation, increased mucus secretion, and bronchial hyperreactivity.^1^ There is growing appreciation that asthma is a heterogeneous disease comprised of different phenotypes driven by distinct pathophysiological mechanisms.^2^ Many asthmatic patients have evidence of increased type 2 inflammation (type 2‐high), which is characterized by airway eosinophilia and elevated T helper 2 (Th2) cytokines, such as interleukin‐4 (IL‐4), IL‐5, and IL‐13.^3^ However, a subset of asthma patients (type 2‐low) have low or absent airway eosinophils and lack other biomarkers of type 2 inflammation.^4^ A number of patients with type 2‐low asthma have neutrophilic airway inflammation that is poorly responsive to treatment with glucocorticoids.^4^, ^5^, ^6^ While the mechanisms driving neutrophilic asthma are incompletely understood, activation of innate signaling pathways in the respiratory tract by pathogens or pollutants likely contributes to disease pathogenesis.^5^, ^7^, ^8^ Animal models have shown that sensitization to inhaled antigens in combination with Toll‐like receptor (TLR) ligands results in Th1‐ and/or Th17‐driven neutrophilic inflammation following antigen challenge.^9^, ^10^, ^11^ In humans, neutrophilic asthma has been associated with both increased Th1 and Th17 responses.^12^ Furthermore, defects in the clearance of apoptotic neutrophils by airway macrophages, a process termed efferocytosis, may also contribute to persistent airway neutrophilia.^13^ While several biological agents are now available for the treatment of type 2‐high asthma, effective therapies for type 2‐low asthma, including those with neutrophilic airway inflammation, remain elusive.^14^ Identifying the key molecular mechanisms that regulate neutrophilic inflammatory responses to inhaled antigens may reveal novel therapeutic targets for type 2‐low asthma.

Neuropilins are a family of multifunctional proteins that play important roles in neuronal development, angiogenesis, and immunity.^15^ The two family members, neuropilin‐1 (NRP1) and neuropilin‐2 (NRP2), have both transmembrane and soluble forms that are important in regulating immune responses.^16^ While the immunoregulatory function of NRP1 has been well characterized, relatively little is known regarding the role of NRP2 in the immune system.^17^ We previously reported that NRP2 was expressed by murine and human alveolar macrophages (AM) and was upregulated in response to lipopolysaccharide (LPS).^18^ Importantly, myeloid‐specific ablation of NRP2 resulted in prolonged airway neutrophilia following inhaled LPS challenge in mice, indicating that NRP2 regulates TLR‐mediated inflammatory responses in the lungs.^18^ Because TLR responses have been implicated in the pathogenesis of type 2‐low asthma, we investigated the role of NRP2 in a murine model of neutrophilic asthma. We found that TLR ligands, but not Th2‐promoting adjuvants, induce NRP2 expression by AM. NRP2 expression by AM and other lung antigen‐presenting cells was increased in mice with neutrophilic asthma. Moreover, myeloid‐specific ablation of NRP2 resulted in increased inflammatory cell numbers in the airways of mice with neutrophilic but not eosinophilic asthma, suggesting that NRP2 is a specific regulator of type 2‐low inflammation. Myeloid‐specific ablation of NRP2 did not affect Th1/Th17 responses to inhaled antigens, but rather resulted in impaired AM efferocytosis. Taken together, our findings suggest that NRP2 expression by AM is a negative regulator of neutrophilic asthma.

## 2 MATERIALS AND METHODS

### 2.1 Mice

C57BL/6J, *Nrp2* ^*gfp/+*^ (*Nrp2* ^*tm1.2Mom*^/MomJ), and LysMcre (B6.129P2‐*Lyz2* ^*tm1(cre)Ifo*^/J) mice were obtained from Jackson Laboratories. *Nrp2* ^*fl/fl*^ (*Nrp2* ^*tm1.1Mom*^/MomJ) mice were kindly provided by Tracy Tran (Rutgers University). LysMcre/*Nrp2* ^*fl/fl*^ were generated by crossing LysMcre and *Nrp2* ^*fl/fl*^ as previously described.^18^ All mice were housed in specific pathogen‐free conditions and used between 6 and 12 weeks of age. All animal experiments were approved by the Institutional Animal Care and Use Committee at the University of North Carolina at Chapel Hill (UNC‐Chapel Hill).

### 2.2 Isolation and ex vivo treatment of murine AM

Murine AM was isolated by bronchoalveolar lavage (BAL) as previously described^18^ and resuspended in complete Dulbecco's modified Eagle's medium (DMEM) (DMEM, 10% fetal bovine serum [Gemini], penicillin/streptomycin). AM was seeded into 96‐well plates at 10^5^ cells/well and treated with 100 ng/ml of ultrapure LPS from *Escherichia coli* O111:B4 (InvivoGen), 1 μg/ml Pam3CSK4 (Invivogen), 20 μg/ml *Aspergillus oryzae* protease (ASP; Sigma‐Aldrich), or 1% (vol/vol) house dust extract (HDE) prepared as previously described.^19^ After 6 h, AM were collected and total RNA was isolated for analysis.

### 2.3 Mouse models of neutrophilic and eosinophilic asthma

For the neutrophilic asthma model, mice were lightly anesthetized by isoflurane inhalation and administered 50 μg of LPS‐free ovalbumin (OVA) (Worthington Biomedical) alone or in combined with 100 ng LPS from *Escherichia coli* O111:B4 (Sigma‐Aldrich) by oropharyngeal (op) aspiration on Days 0 and 7. For the eosinophilic asthma model, lightly anesthetized mice were administered 50 μg of LPS‐free OVA alone or combined with 20 μg of ASP (Sigma‐Aldrich) by op aspiration on Days 0 and 7 as previously described.^20^ For both models, mice were challenged daily on Days 14–16 with op administration of 50 μg of OVA (Grade V; Sigma‐Aldrich). On Day 17, mice were euthanized and bronchoalveolar lavage fluid (BALF) was collected for cytology and measurement of cytokines or the soluble isoform of NRP2 (sNRP2) by enzyme‐linked immunosorbent assay (ELISA) as previously described.^18^ For histological studies, lungs were fixed in 10% buffered formalin, sectioned and stained with periodic acid‐Schiff (PAS) stain, and analyzed by microscopy.

### 2.4 Measurement of airway hyperresponsiveness

Mice were anesthetized with pentobarbital, tracheostomized, and paralyzed with atracurium. Mice were then mechanically ventilated with a computer‐controlled ventilator (Scireq) at 300 breaths/min, with a tidal volume of 6 ml/kg and a positive end‐expiratory pressure of 3 cm. Baseline resistance and bronchial responsiveness to inhaled aerosolized methacholine were assessed with 10 s challenges of 25 and 50 mg/ml methacholine (Sigma‐Aldrich), as previously described.^21^

### 2.5 Lymph node restimulation assay

Mice were sensitized and challenged with OVA as described above. On Day 17, mediastinal lymph nodes (mLNs) were harvested and passed through a 70 μm strainer to obtain a single‐cell suspension. Lymph node (LN) cells (200,000 cells/well) were cultured in complete Roswell Park Memorial Institute (RPMI) medium (RPMI‐1640, 10% fetal bovine serum [Gemini], penicillin–streptomycin, 50 μM 2‐mercaptoethanol, and 10 mM HEPES) and OVA (10 μg/ml) in a 96‐well round‐bottom plate at 37°C in a 5% CO_2_ incubator. Supernatants were collected 4 days later and cytokines were measured by ELISA.

### 2.6 Flow cytometry analysis of murine lung leukocyte populations

Murine lung leukocytes were isolated and analyzed by flow cytometry as previously described.^19^, ^22^ Briefly, harvested lungs were minced and digested with Liberase TM (100 μg/ml; Roche), collagenase XI (250 μg/ml), hyaluronidase 1a (1 mg/ml), and DNase I (200 μg/ml; Sigma‐Aldrich) for 1 h at 37°C. The digested tissue was passed through a 70 µm nylon strainer to obtain a single‐cell suspension. Red blood cells were lysed with 0.15 M ammonium chloride and 1 mM potassium bicarbonate. For antibody staining of surface antigens, cells were incubated with anti‐mouse CD16/CD32 (2.4G2) for 5 min to block Fc receptors, followed by incubation with fluorochrome‐ or biotin‐conjugated antibodies against murine CD3ε (145‐2C11), CD11b (M1/70), CD11c (N418), CD19 (6D5), CD88 (20/70), CD103 (M290), Ly‐6C (AL‐21), Ly‐6G (1A8), I‐A/E (M5/114.15.2) (BioLegend); or Siglec‐F (E50‐2440) (BD Biosciences) for 30 min on ice. Staining with biotinylated antibodies was followed by incubation with fluorochrome‐conjugated streptavidin for 20 min on ice. Cells were also concurrently stained with Zombie Aqua^TM^ (BioLegend) for live cell/dead cell discrimination. Flow cytometry data were acquired with four laser LSRII (BD Biosciences) and analyzed using FlowJo (Treestar) software. Only single cells were analyzed. Lung macrophage and dendritic cell (DC) subpopulations were identified as follows: AM (CD45^+^CD11c^hi^Siglec‐F^hi^), interstitial macrophages (IM; CD45^+^CD88^hi^CD11b^hi^I‐A/E^hi^Ly‐6C^lo^), monocytes (CD45^+^CD88^hi^CD11b^hi^I‐A/E^lo^Ly‐6C^hi^), inflammatory monocyte‐derived macrophages (Inflamm Macs; CD45^+^CD88^hi^CD11b^hi^I‐A/E^hi^Ly‐6C^hi^), CD103^+^ conventional DCs type 1 (CD45^+^Siglec‐F^lo^CD11c^hi^I‐A/E^hi^Ly‐6C^lo^CD11b^lo^CD103^hi^), and CD11b^+^ conventional DCs type 2 (CD45^+^Siglec‐F^lo^CD11c^hi^I‐A/E^hi^Ly‐6C^lo^CD11b^hi^CD103^lo^).

### 2.7 Generation of NRP2‐deficient RAW 264.7 macrophages

Cas9‐expressing RAW 264.7 cells (RAW‐Cas9) were a generous gift of Rob Hagan (UNC‐Chapel Hill). Single‐guide RNAs (Synthego) targeting exon 2 of the *Nrp2* gene were electroporated into RAW‐Cas9 cells using a Neon transfection system (Thermo Fisher Scientific). CRISPR editing of the *Nrp2* gene was confirmed using the Surveyor® Mutation Detection Kit (IDT). Clones of transfected RAW‐Cas9 cells were generated by limiting dilution and screened for deletion of NRP2 protein expression by flow cytometry and immunoblotting. A clone (NRP2KO‐RAW) expressing <2% NRP2 protein relative to RAW‐Cas9 cells was identified and mutagenesis of exon 2 in both *Nrp2* alleles was confirmed by Sanger sequencing.

### 2.8 Efferocytosis assays

To generate apoptotic cells, Jurkat cells were treated with 1 µM staurosporine (Thermo Fisher Scientific) for 4 h, which resulted in >90% of cells being positive for annexin‐V and negative for 7‐aminoactinomycin D staining as determined by flow cytometry. Apoptotic Jurkat cells were washed and then labeled with the PKH26 Red Fluorescent Cell Linker Kit (Sigma‐Aldrich) as per the manufacturer's instructions. PKH26‐labeled apoptotic Jurkat cells were cultured with adherent AM or RAW 264.7 macrophages at a ratio of 5:1 apoptotic cells to phagocytes at 37°C. After 2 h, the adherent phagocytes were washed vigorously to remove free or noninternalized apoptotic cells bound to the cell surface. The phagocytes were then harvested and analyzed by flow cytometry to quantify efferocytosis of PKH26‐labeled apoptotic Jurkat cells. The level of efferocytosis was evaluated by calculating the phagocytic index as previously described: phagocytic index = percent of PKH26^+^ cells × mean fluorescence intensity of PKH26^+^ phagocytes.^23^ In some experiments, AM were treated with apoptotic cells for 24 h, after which supernatant were collected and levels of IL‐10 and transforming growth factor‐β (TGF‐β) were measured by ELISA.

### 2.9 Complementary DNA amplification and analysis

Total RNA was isolated from cells or lung tissue using TRIzol reagent (Life Technologies) and converted to complementary DNA with oligo(dT) and random hexamer primers using MuLV reverse transcriptase (Life Technologies). Quantitative polymerase chain reaction (qPCR) was performed using PerfeCTa SYBR Green PCR Mastermix (Quantabio) and a 7300 Real‐Time PCR System (Applied Biosystems). KiCqStart® SYBR® Green Primers (Sigma‐Aldrich) specific for murine *Nrp2, Cxcl1, Cxcl2, Cxcl5*, or *Icam1*. The efficiency‐corrected Δ*C*_t_ for each gene was determined and normalized to *Actb*.

### 2.10 Statistical analysis

Data are expressed as means ± SEM. Statistical differences between groups were calculated using a two‐tailed Student's *t*‐test or one‐way analysis of variance (ANOVA) with a Dunnett multiple comparison test. For airway hyperactivity (AHR) studies, differences between groups were determined by two‐way ANOVA with a Tukey multiple comparison test. For all analyses, a *p* < .05 was considered significant. Statistical analyses were performed using GraphPad Prism 8 software.

## 3 RESULTS

### 3.1 NRP2 expression is increased in a neutrophilic asthma model

**Figure 1:**
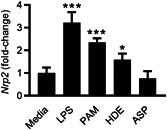
TLR ligands, but not proteases, induce *Nrp2* expression in murine AM. AM from C57BL/6J mice was treated with ultrapure LPS (TLR4 ligand), Pam3CSK4 (TLR2 ligand), house dust extract (HDE), or *Aspergillus oryzae* protease (ASP). Six hours later, the fold‐change of *Nrp2* mRNA expression (relative to AM treated with media alone) was determined by qPCR. Bars represent mean ± SEM (*n* = 3 mice per group). *p < .05, ***p < .001, one‐way analysis of variance. AM, alveolar macrophages; LPS, lipopolysaccharide; mRNA, messenger RNA; NRP2, neuropilin‐2; PAM, Pam3CSK4; qPCR, quantitative polymerase chain reaction; TLR, Toll‐like receptor

Because they direct the type of immune responses elicited against inhaled antigens, environmental adjuvants such as TLR ligands and proteases are important determinants of asthma phenotypes.^7^ Exposure to TLR ligands can promote Th1/Th17 responses against inhaled antigens, resulting in neutrophilic airway inflammation.^11^, ^24^ In contrast, proteases prime Th2 responses against inhaled antigens, leading to a type 2‐high eosinophilic inflammatory response.^20^, ^25^ AM plays an important role in recognizing inhaled environmental adjuvants and controlling airway inflammation through the expression of immunoregulatory molecules,^26^ including NRP2.^18^ To determine if environmental adjuvants differentially induce *Nrp2* in AM, we treated murine AM with TLR ligands or proteases. Treatment with either LPS (TLR4 ligand) or Pam3CSK4 (TLR2 agonist) induced *Nrp2* expression in AM (Figure 1). Exposure to HDE, which contains a mixture of TLR ligands and proteases,^20^ also induced *Nrp2* expression in AM (Figure 1). However, exposure to ASP, a potent Th2 adjuvant,^20^ failed to induce *Nrp2* expression in AM (Figure 1). Thus, TLR ligands, but not Th2‐promoting proteases, stimulate *Nrp2* expression in AM.

**Figure 2:**
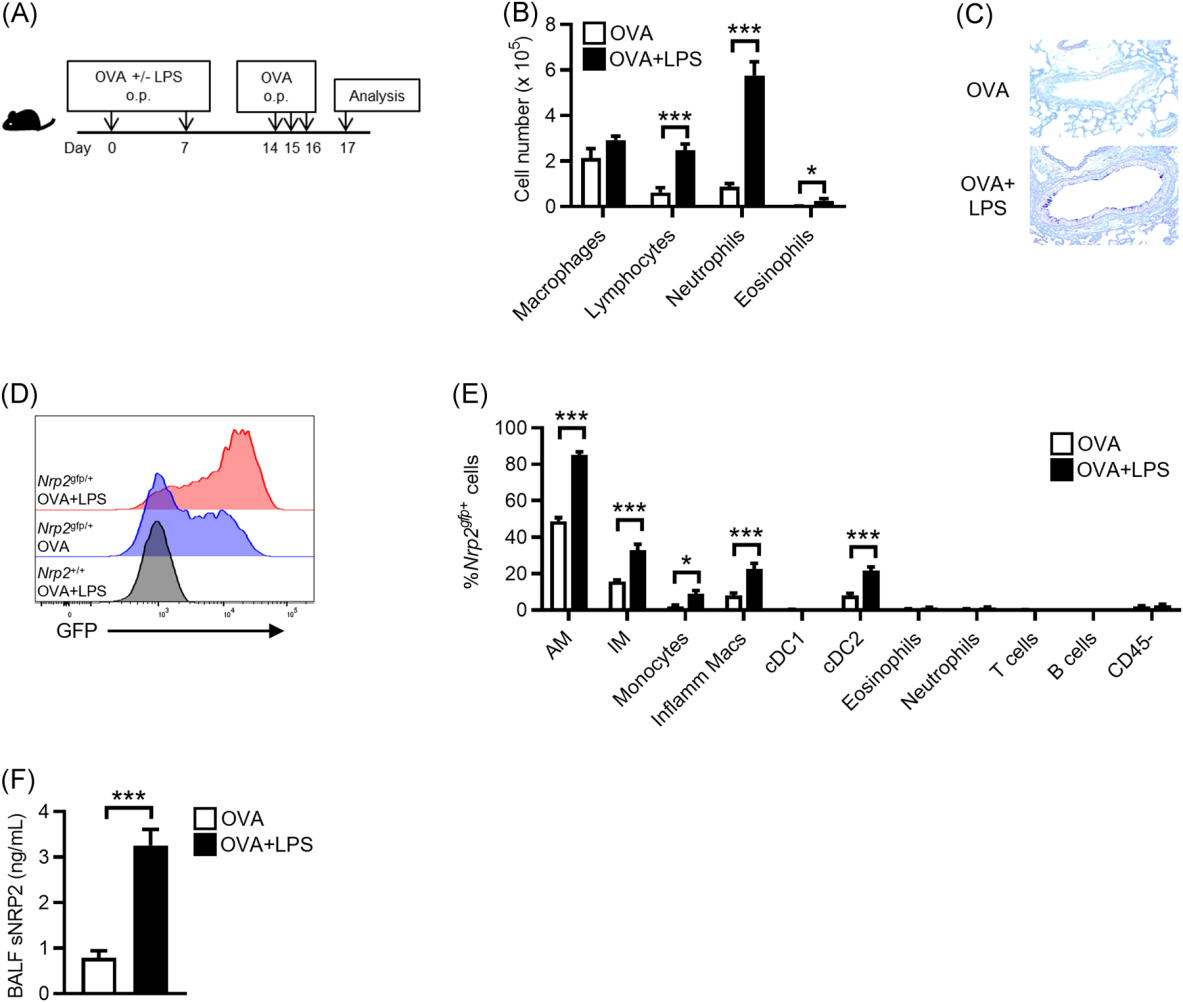
Lung NRP2 expression is increased in a murine model of neutrophilic asthma. (A) Schematic of neutrophilic asthma model. (B) Cell differentials in bronchoalveolar lavage fluid (BALF) following ovalbumin (OVA) challenge of C57BL/6J mice sensitized with either OVA alone (white bars) or OVA + LPS (black bars). Bars represent means ± SEM (*n* = 7 mice per group). (C) Representative periodic acid‐Schiff staining of airways from OVA‐challenged C57BL/6J mice sensitized with either OVA or OVA + LPS. (D) Representative cytograms showing *Nrp2* ^*gfp*^ expression by AM from *Nrp2* ^*gfp*/+^ reporter mice sensitized with either OVA (blue histogram) or OVA + LPS (red histogram). AM from wild‐type (*Nrp2* ^+/+^) mice was included as a negative control for gating (black histogram). (E) Quantitation of *Nrp2* ^*gfp*^ expression by lung cells from OVA‐challenged *Nrp2* ^*gfp*/+^ reporter mice (*n* = 5–7 mice per group) sensitized with either OVA (white bars) or OVA + LPS (black bars). Bars represent means ± SEM (*n* = 5–7 mice per group). Combined data from two independent experiments are presented. (F) Measurement of soluble NRP2 (sNRP2) in BALF from OVA‐challenged C57BL6/J mice sensitized with OVA (white bars) or OVA + LPS (black bars) by ELISA. Bars represent mean ± SEM (*n* = 7 mice per group). *p < .05, ***p < .001, Student's *t*‐test. (F) Measurement of sNRP2 in BALF from OVA‐challenged C57BL6/J mice sensitized with OVA (white bars) or OVA + LPS (black bars) by ELISA. Bars represent mean ± SEM (*n* = 7 mice per group). *p < .05, ***p < .001, Student's *t*‐test. AM, alveolar macrophages; ELISA, enzyme‐linked immunosorbent assay; GFP, green fluorescent protein; IM, interstitial macrophages; Inflamm Macs, inflammatory monocyte‐derived macrophages; LPS, lipopolysaccharide; NRP2, neuropilin‐2; ns, not significant; op, oropharyngeal

Because exposure to TLR ligands has been associated with the development of neutrophilic inflammation against inhaled antigens,^11^ we investigated if *Nrp2* expression is altered in a murine model of neutrophilic asthma. In this model, mice were sensitized via the airways to OVA alone or in combination with LPS (OVA + LPS) on Days 0 and 7. Mice were then challenged daily with airway administration of OVA alone on Days 14–16, and airway inflammation was assessed by BAL and lung histology on Day 17 (Figure 2A). As expected, mice sensitized to OVA alone did not develop airway inflammation following the OVA challenge (Figure 2B). In contrast, mice exposed to OVA + LPS developed a significant increase in airway neutrophils and lymphocytes (Figure 2B). Histological analysis showed increased PAS^+^ cells in the airways of mice receiving OVA + LPS, consistent with goblet cell metaplasia (Figure 2C). Thus, sensitization of mice with OVA + LPS resulted in a phenotype consistent with neutrophilic asthma.

We next investigated if *Nrp2* expression was altered in the lungs of mice with neutrophilic asthma. For this, we used heterozygous *Nrp2* ^*gfp/+*^ reporter mice in which GFP fluorescence reflects *Nrp2* expression.^18^ *Nrp2* ^*gfp/+*^ mice were sensitized to either OVA or OVA + LPS and then challenged with inhaled OVA. Following OVA challenge, lungs were harvested and *Nrp2* ^*gfp*^ expression by lung leukocyte populations was analyzed by multicolor flow cytometry (see Figure S1 for gating strategy). In mice sensitized with OVA alone, *Nrp2* ^*gfp*^ expression was detected in a small percentage of AM, IM, monocytes, Inflamm Macs, and CD11b^+^ conventional DCs type 2 (cDC2s) (Figure 2D,E). In contrast, mice sensitized with OVA + LPS had significantly higher percentages of AM, IM, Inflamm Macs, monocytes, and cDC2s expressing *Nrp2* ^*gfp*^ following the OVA challenge (Figure 2D,E). We did not observe significant *Nrp2* ^*gfp*^ expression in CD103^+^ conventional cDC1s, neutrophils, eosinophils, or lymphocytes from mice sensitized with either OVA alone or OVA + LPS (Figure 2E). Thus, *Nrp2* expression is induced in lung macrophages, monocytes, and cDC2s in mice with neutrophilic asthma.

We previously reported that an sNRP2 was increased in the airways of mice following acute exposure to inhaled LPS, likely due to ectodomain shedding of transmembrane NRP2 by AM.^18^ We, therefore, investigated if airway levels of sNRP2 were similarly increased in mice with neutrophilic asthma. Compared to sensitization with OVA alone, mice sensitized with OVA + LPS had significantly increased levels of sNRP2 in BALF following the OVA challenge (Figure 2F). In summary, both *Nrp2* expression and sNRP2 levels are increased in the lungs of mice with neutrophilic asthma.

### 3.2 Myeloid‐specific ablation of NRP2 augments airway inflammation in a neutrophilic asthma model

**Figure 3:**
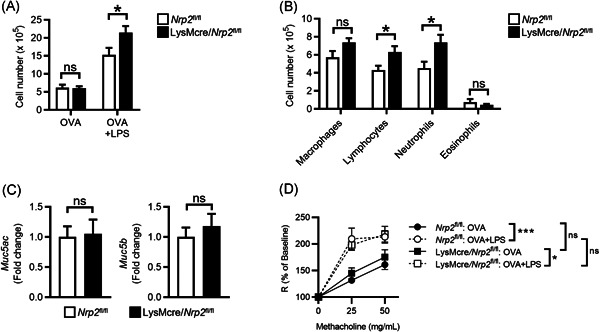
LysMcre/*Nrp2* ^*fl/fl* ^mice have enhanced airway inflammation in a neutrophilic asthma model. (A) Total BALF cell counts in *Nrp2* ^*fl*/*fl*^ (white bars) or LysMcre/*Nrp2* ^*fl*/*fl*^ mice (black bars) sensitized with either OVA or OVA + LPS and then challenged with OVA as in Figure 2A. Bars represent mean ± SEM (*n* = 4–6 mice for OVA only groups; *n* = 14–16 for OVA + LPS groups). *p < .05, ***p < .001, Student's *t*‐test. (B, C) BALF cell differential counts (B) and lung mucin gene expression (C) from OVA‐challenged *Nrp2* ^*fl*/*fl*^ or LysMcre/*Nrp2* ^*fl*/*fl*^ mice sensitized with OVA + LPS. Bars represent mean ± SEM (*n* = 14–16 mice per group). (D) Airway resistance (*R*) following methacholine challenge of mice treated as indicated. Symbols represent mean ± SEM (*n* = 5 mice for OVA only groups; *n* = 8 for OVA + LPS groups). *p < .05, ***p < .001, two‐way ANOVA with Tukey multiple comparison tests. ANOVA, analysis of variance; BALF, bronchoalveolar lavage fluid; LPS, lipopolysaccharide; NRP2, neuropilin‐2; ns, not significant; OVA, ovalbumin

We previously reported that myeloid‐specific expression of *Nrp2* suppressed acute inflammatory responses to inhaled LPS.^18^ Our finding that *Nrp2* expression was increased in macrophages from mice sensitized with OVA + LPS suggested that NRP2 may also have an immunoregulatory role in neutrophilic asthma. To investigate this possibility, we examined airway inflammation in LysMcre/*Nrp2* ^*fl/fl*^ mice, which lack expression of *Nrp2* in lung macrophages.^18^ in our neutrophilic asthma model. BALF total cell counts were similar between *Nrp2* ^*fl/fl*^ control mice and LysMcre/*Nrp2* ^*fl/fl*^ sensitized to OVA alone and challenged with OVA, indicating that myeloid‐specific ablation did not enhance sensitization to antigen in the absence of an adjuvant (Figure 3A). In contrast, LysMcre/*Nrp2* ^*fl/fl*^ mice sensitized with OVA + LPS demonstrated a significant increase in BALF total cell number following OVA challenge compared to *Nrp2* ^*fl/fl*^ mice (Figure 3A). BALF cell differential analysis of mice sensitized with OVA + LPS revealed that airway neutrophils and lymphocytes were significantly increased in LysMcre/*Nrp2* ^*fl/fl*^ mice compared to *Nrp2* ^*fl/fl*^ mice (Figure 3B). PAS staining of lungs revealed that goblet cell metaplasia was similar between LysMcre/*Nrp2* ^*fl/fl*^ and *Nrp2* ^*fl/fl*^ mice following OVA challenge (data not shown). To better quantify mucus production, we also analyzed mucin expression in the lungs following the OVA challenge by qPCR and found similar levels of *Muc5ac* and *Muc5b* messenger RNA (mRNA) in LysMcre/*Nrp2* ^*fl/fl*^ and *Nrp2* ^*fl/fl*^ mice (Figure 3C). We next investigated if AHR was increased in LysMcre/*Nrp2* ^*fl/fl*^ mice with neutrophilic asthma. As expected, sensitization with OVA + LPS resulted in a significant increase in airway resistance following the OVA challenge compared to sensitization with OVA alone for both LysMcre/*Nrp2* ^*fl/fl*^ and *Nrp2* ^*fl/fl*^ mice (Figure 3D). However, airway resistance was similar in LysMcre/*Nrp2* ^*fl/fl*^ and *Nrp2* ^*fl/fl*^ mice sensitized with OVA + LPS (Figure 3D), indicating that myeloid specific‐ablation of NRP2 did not affect AHR in our model. Taken together, our findings indicate that myeloid‐specific ablation of NRP2 results in enhanced airway inflammation, but not AHR or mucus production, in mice with neutrophilic asthma.

### 3.3 Myeloid‐specific ablation of NRP2 does not exacerbate airway inflammation in an eosinophilic asthma model

**Figure 4:**
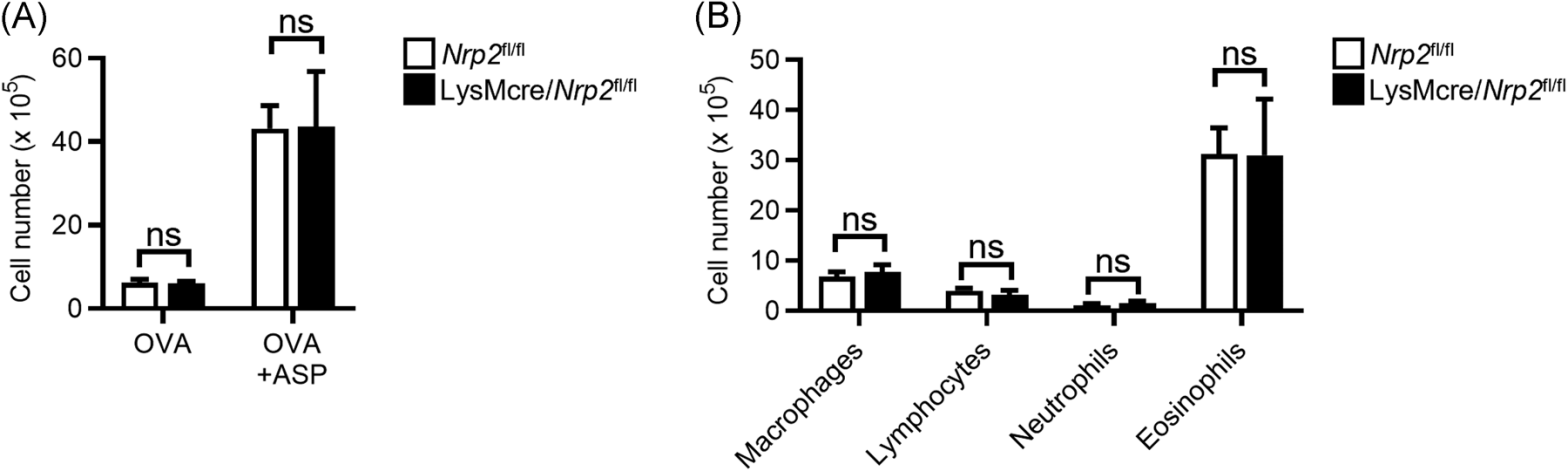
Myeloid cell expression of NRP2 does not regulate inflammation in an eosinophilic asthma model. (A) BALF total cell counts and cell differential counts in *Nrp2* ^*fl*/*fl*^ (white bars) or LysMcre/*Nrp2* ^*fl*/*fl*^ mice (black bars) sensitized with either OVA or OVA + ASP and then challenged with OVA. Bars represent mean ± SEM (*n* = 5‐7 mice per group). (B) BALF cell differential counts from OVA‐challenged *Nrp2* ^*fl*/*fl*^ or LysMcre/*Nrp2* ^*fl*/*fl*^ mice sensitized with OVA + ASP. Bars represent mean ± SEM (*n* = 6 mice per group). ASP, *Aspergillus oryzae* protease; BALF, bronchoalveolar lavage fluid; NRP2, neuropilin‐2; ns, not significant; OVA, ovalbumin

Our findings indicated that myeloid‐specific expression of NRP2 negatively regulated inflammatory responses to inhaled allergens, but whether this was specific to type 2‐low neutrophilic inflammation was unclear. We thus investigated if the myeloid‐specific expression of NRP2 also regulated Th2‐mediated airway inflammation using a protease‐induced eosinophilic asthma model. Mice were sensitized via the airways to OVA alone or in combination with the Th2 adjuvant ASP, which results in eosinophilic airway inflammation upon OVA challenge.^20^ LysMcre/*Nrp2* ^*fl/fl*^ and *Nrp2* ^*fl/fl*^ mice sensitized with OVA + ASP had equivalent numbers of total airway leukocytes and eosinophils following OVA challenge (Figure 4A,B). We also did not observe differences in goblet cell metaplasia in LysMcre/*Nrp2* ^*fl/fl*^ and *Nrp2* ^*fl/fl*^ mice as measured by PAS staining of lungs (data not shown). Thus, NRP2 appears to regulate neutrophilic, but not eosinophilic, inflammatory responses to inhaled allergens.

### 3.4 Myeloid‐specific ablation of NRP2 does not affect T helper cell responses to inhaled antigens or lung expression of neutrophil recruitment factors

**Figure 5:**
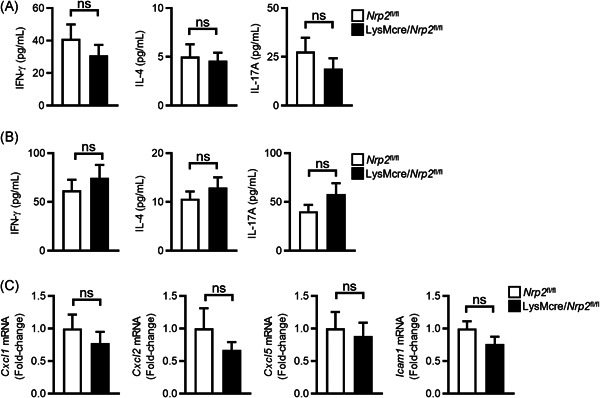
Myeloid‐specific ablation of NRP2 does not affect T helper cell responses to inhaled antigens or lung expression of neutrophil recruitment factors. (A, B) *Nrp2* ^*fl*/*fl*^ (white bars) or LysMcre/*Nrp2* ^*fl*/*fl*^ mice (black bars) were sensitized with OVA and LPS and then challenged with OVA as in Figure 2A. (A) Lung‐draining lymph nodes cells were collected following the OVA challenge and stimulated ex vivo with OVA antigen. Four days later, levels of T helper cytokines (IFN‐γ, IL‐4, and IL‐17A) in cell culture supernatants were measured by ELISA. (B) T helper cytokine levels in BALF following OVA challenge as measured by ELISA. (C) Lung expression of neutrophil chemokines and ICAM‐1 as determined by qPCR. Data are presented as fold‐change relative to *Nrp2* ^*fl*/*fl*^ mice. Bars represent mean ± SEM (*n* = 11–16 mice per group), Student's *t*‐test. BALF, bronchoalveolar lavage fluid; ELISA, enzyme‐linked immunosorbent assay; IFN‐γ, interferon‐γ; IL‐4, interleukin‐4; LPS, lipopolysaccharide; mRNA, messenger RNA; NRP2, neuropilin‐2; ns, not significant; OVA, ovalbumin

Detrimental Th1 and/or Th17 responses to inhaled antigens have been implicated in the pathogenesis of neutrophilic asthma.^12^ To determine if myeloid‐specific ablation enhances Th1/Th17 priming to inhaled antigen, we collected mLNs from LysMcre/*Nrp2* ^*fl/fl*^ and *Nrp2* ^*fl/fl*^ mice that were sensitized to OVA + LPS and then challenged with OVA antigen. LN cells were restimulated ex vivo with OVA and T helper cytokine production was measured by ELISA. We did not observe differences in the production of Th1 (interferon‐γ [IFN‐γ]), Th2 (IL‐4), or Th17 (IL‐17A) cytokines (Figure 5A), indicating that antigen‐specific T cell priming was not altered in LysMcre/*Nrp2* ^*fl/fl*^ mice. We also did not observe differences in T helper cytokines in BALF from LysMcre/*Nrp2* ^*fl/fl*^ and *Nrp2* ^*fl/fl*^ mice following the OVA challenge (Figure 5B), indicating that myeloid‐specific ablation of NRP2 did not alter effector T helper cell responses in the lungs. Thus, the enhanced airway inflammation observed in LysMcre/*Nrp2* ^*fl/fl*^ mice was unlikely due to increased Th1/Th17 responses to inhaled antigens.

Chemokines that signal though CXCR2 (e.g., CXCL1, CXCL2, and CXCL5) are important for the recruitment of neutrophils to inflamed airways.^27^ Additionally, the expression of the leukocyte adhesion molecule ICAM‐1 on lung endothelial cells has been reported to play a critical role in neutrophil recruitment to the lungs in response to inflammatory stimuli.^28^ We, therefore, measured the expression of neutrophil chemokines and ICAM‐1 in the lungs of LysMcre/*Nrp2* ^*fl/fl*^ and *Nrp2* ^*fl/fl*^ mice that were sensitized to OVA + LPS and then challenged with OVA antigen. We did not observe differences in expression of *Cxcl1, Cxcl2, Cxcl5*, or *Icam1* in lungs from LysMcre/*Nrp2* ^*fl/fl*^ and *Nrp2* ^*fl/fl*^ mice (Figure 5C). Thus, increased expression of neutrophilic recruitment factors is unlikely responsible for the increased airway inflammation observed in LysMcre/*Nrp2* ^*fl/fl*^ mice.

### 3.5 Efferocytosis is impaired in NRP2‐deficient AM

**Figure 6:**
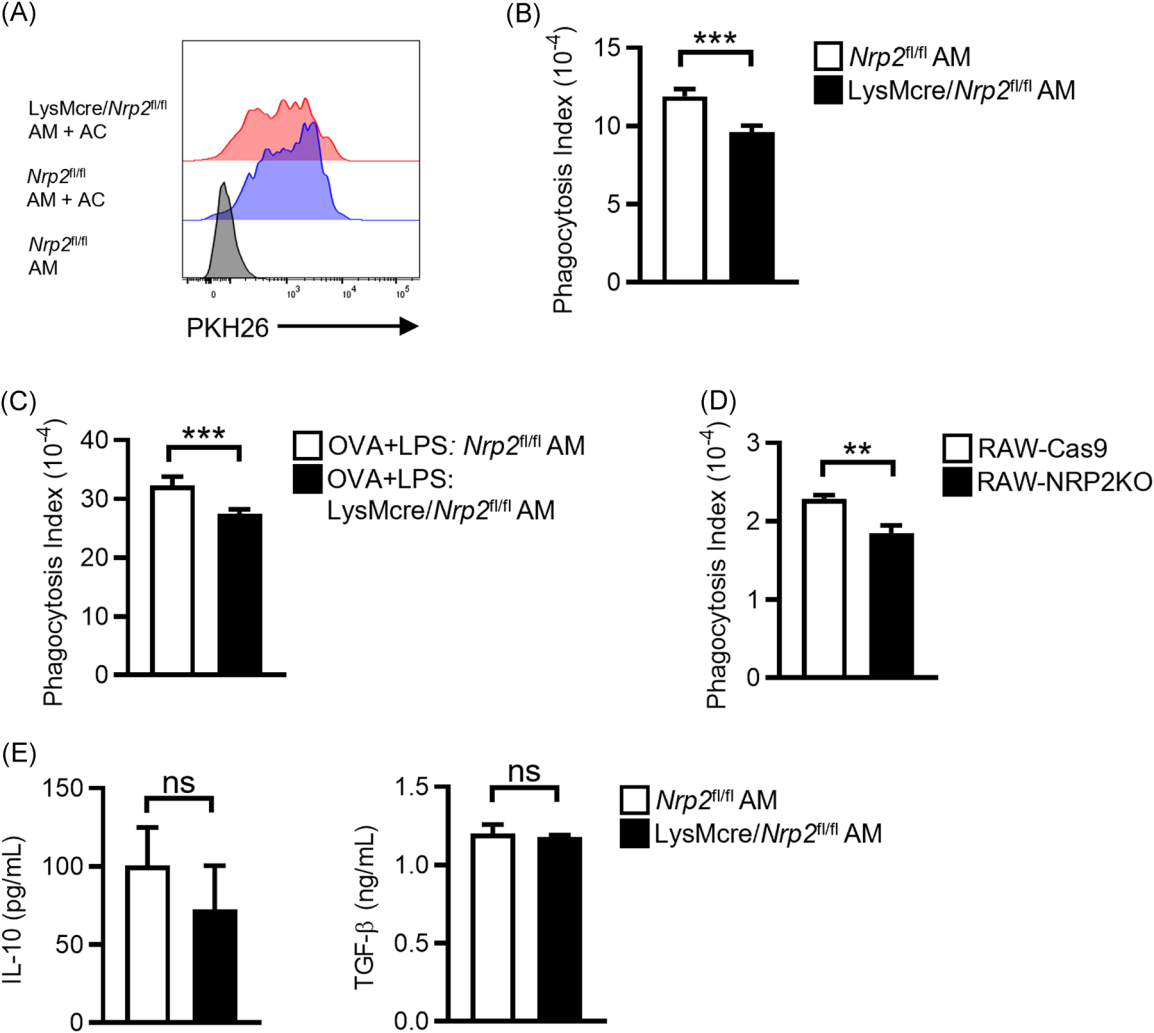
Efferocytosis is impaired in NRP2‐deficient AM. (A, B) AM from *Nrp2^fl/fl^* or LysMcre/*Nrp2^fl/fl^* mice were cultured with PKH26‐labeled apoptotic Jurkat cells at a ratio of 5:1 apoptotic cells:AM. Two hours later, AM was washed and efferocytosis of apoptotic cells (AC) was determined by flow cytometry. (A) Representative cytograms showing efferocytosis of PKH26‐labeled apoptotic cells by AM from *Nrp2* ^fl/fl^ mice (blue histogram) or LysMcre/*Nrp2* ^fl/fl^ mice (red histogram). AM cultured without apoptotic cells (gray histogram) were included as a negative control for gating. (B) Phagocytosis index for *Nrp2^fl/fl^* or LysMcre/*Nrp2^fl/fl^* AM. Bars represent mean ± SEM ( = 5 mice per group). (C) AM was isolated from *Nrp2^fl/fl^* or LysMcre/*Nrp2^fl/fl^* mice that had been sensitized with OVA + LPS and challenged with OVA as in Figure 2A. Efferocytosis of apoptotic Jurkat cells was determined as in (A). Bars represent mean ± SEM (*n* = 5 mice per group). (D) Phagocytosis index for NRP2‐deficient (RAW‐NRP2KO) and control (RAW‐Cas9) RAW 264.7 macrophages. Bars represent mean ± SEM of triplicate wells. Representative data from one of two independent experiments is shown. (E) AM from *Nrp2^fl/fl^* or LysMcre/*Nrp2^fl^ ^/fl^* mice were cultured with apoptotic cells at a ratio of 5:1 apoptotic cells:AM for 24 h. Levels of IL‐10 and TGF‐β in cell culture supernatants were measured by ELISA. **p < .01, ***p < .001, Student's *t‐*test. AM, alveolar macrophages; ELISA, enzyme‐linked immunosorbent assay; IL‐10, interleukin‐10; LPS, lipopolysaccharide; NRP2, neuropilin‐2; ns, not significant; OVA, ovalbumin; TGF‐β, transforming growth factor‐β

Efferocytosis of apoptotic inflammatory cells by macrophages is essential for the resolution of airway inflammation.^29^ Impaired efferocytosis by AM has been associated with severe or steroid‐refractory asthma in humans.^13^, ^30^, ^31^ NRP2 was reported to regulate efferocytosis by tumor‐associated macrophages,^32^ but whether it has a similar role in AM is unknown. To investigate this, we evaluated efferocytosis by NRP2‐deficient AM collected from LysMcre/*Nrp2* ^*fl/fl*^ mice. Efferocytosis of apoptotic Jurkat cells labeled with the fluorescent dye PKH26 was significantly decreased in LysMcre/*Nrp2* ^*fl/fl*^ AM compared to *Nrp2* ^*fl/fl*^ AM (Figure 6A,B). To determine if AM efferocytosis was also impaired in mice with neutrophilic asthma, we isolated AM from the airways of *Nrp2* ^*fl/fl*^ and LysMcre/*Nrp2* ^*fl/fl*^ mice that had been sensitized with OVA + LPS and then challenged with OVA via the airways. Similar to our results with AM from untreated mice, AM from LysMcre/*Nrp2* ^*fl/fl*^ mice with neutrophilic asthma exhibited impaired efferocytosis of apoptotic Jurkat cells (Figure 6C). To further verify the role of NRP2 in efferocytosis, we generated NRP2‐deficient RAW 264.7 macrophages (RAW‐NRP2KO) using CRISPR‐Cas9 genome editing. Similar to our findings with NRP2‐deficient AM, efferocytosis of PKH26‐labeled apoptotic cells was decreased in RAW‐NRP2KO cells compared to RAW‐Cas9 control cells (Figure 6D).

Efferocytosis of apoptotic cells by macrophages can induce the production of immunosuppressive cytokines such as IL‐10 and TGF‐β.^33^, ^34^ Deletion of NRP2 in tumor‐associated macrophages was reported to result in decreased production of IL‐10 and TGF‐β following exposure to apoptotic cells.^32^ To determine if NRP2 deficiency in AM resulted in a similar finding, we treated LysMcre/*Nrp2* ^*fl/fl*^ or *Nrp2* ^*fl/fl*^ AM with apoptotic cells for 24 h and measured IL‐10 and TGF‐β in cell culture supernatants. We did not observe any significant differences in IL‐10 or TGF‐β production by LysMcre/*Nrp2* ^*fl/fl*^ or *Nrp2* ^*fl/fl*^ AM (Figure 6D), suggesting that NRP2 does not regulate the expression of these cytokines in AM. Taken together, our findings demonstrate that loss of NRP2 impairs efferocytosis of apoptotic cells by AM, which may contribute to the enhanced airway inflammation observed in LysMcre/*Nrp2* ^*fl/fl*^ mice with neutrophilic asthma.

## 4 DISCUSSION

While there have been significant advances in the treatment of patients with eosinophilic asthma, management of patients with type 2‐low asthma, including those with neutrophilic airway inflammation, remains a major clinical challenge.^14^ Patients with neutrophilic asthma tend to have more severe disease that is resistant to glucocorticoid‐based therapies, and, thus, respond poorly to standard asthma treatments.^14^ Interrogating the cellular and molecular mechanisms of neutrophilic asthma is an important prerequisite for the development of effective interventions. Here, we report that the immunoregulatory receptor NRP2 is upregulated in lung macrophages in a murine model of neutrophilic asthma. Myeloid‐specific ablation of NRP2 resulted in enhanced airway inflammation in mice with neutrophilic, but not eosinophilic, asthma. NRP2 did not affect the priming of antigen‐specific T cells or expression of neutrophilic chemotactic factors but was important for efferocytosis by AM. Taken together, these findings suggest that induction of NRP2 in AM limits the severity of airway inflammation associated with neutrophilic asthma.

Exposure to pathogen‐associated molecules such as TLR ligands likely plays an important role in neutrophilic asthma pathogenesis.^5^, ^7^, ^8^ In animal models, coexposure to inhaled allergens and certain TLR ligands (e.g., high‐dose LPS, CpG) during sensitization results in a predominantly neutrophilic inflammatory response following inhaled allergen challenge.^9^, ^24^ In patients with steroid‐resistant asthma, airway colonization with pathogenic bacteria, such as *Moraxella* and *Haemophilus* species, has been associated with more severe disease and neutrophilic airway inflammation.^35^, ^36^ Exposure to pathogen‐derived products such as LPS can stimulate innate immune responses in the respiratory tract, resulting in the production of proinflammatory chemokines that recruit neutrophils to the airways.^37^ Therefore, innate immune responses in the lungs must be tightly regulated to prevent tissue injury. As such, stimulation of innate signaling pathways in AM induces expression of regulatory molecules that inhibit immune signaling pathways and promote resolution of inflammation.^26^ Here, we show that exposure to various TLR ligands induces *Nrp2* expression in AM, whereas exposure to proteases did not. This is consistent with our previous finding that *Nrp2* expression in AM was dependent upon the adaptor molecule MyD88,^18^ which mediates signaling for most TLRs.^38^ The induction of NRP2 in response to TLR ligands but not proteases suggests that NRP2 may specifically regulate neutrophilic rather than eosinophilic inflammatory responses. Indeed, we found that NRP2 was upregulated in lung macrophages in a neutrophilic asthma model, and that lack of NRP2 expression in AM resulted in more severe airway inflammation in mice with neutrophilic, but not eosinophilic, asthma. Interestingly, NRP2 deficiency did not result in increased AHR in mice with neutrophilic asthma, indicating that NRP2 primarily regulates inflammatory responses rather than airway smooth muscle reactivity. While inflammation and airway responsiveness are frequently linked, studies in asthmatics have shown a dissociation between the two processes,^39^ suggesting they may be regulated by distinct mechanisms. Taken together, our findings suggest that induction of NRP2 during neutrophilic asthma is a countermeasure to promote the resolution of airway inflammation.

Studies in humans and mice have revealed a possible role for Th1 and Th17 responses in neutrophilic asthma pathogenesis.^4^ Adoptive transfer of antigen‐specific Th17 cells promotes steroid‐resistant neutrophilic airway inflammation in mice,^40^ and sputum IL‐17A mRNA levels are increased in some patients with neutrophilic asthma.^41^ Others have found that Th1 responses are increased in the airways of patients with steroid‐resistant asthma and that IFN‐γ mediated AHR in a mouse model of severe asthma.^42^ NRP2 is upregulated on M1 polarized macrophages,^16^ which can enhance Th1 and Th17 responses through the production of IL‐12 and IL‐23, respectively.^43^ We, therefore, investigated if myeloid‐specific ablation of NRP2 affected Th1 and Th17 responses in our neutrophilic asthma model. We found that Th1 and Th17 priming in LNs and effector responses in the lungs were similar between LysMcre/*Nrp2* ^*fl/fl*^ and *Nrp2* ^*fl/fl*^ mice, indicating that NRP2 expression by myeloid cells did not regulate antigen‐specific T helper cell responses. We also did not observe an increase in neutrophil chemokine or ICAM‐1 expression in the lungs of LysMcre/*Nrp2* ^*fl/fl*^ mice with neutrophilic asthma, suggesting that NRP2 expression by myeloid cells does not directly regulate neutrophil recruitment to inflamed airways. NRP2 is also expressed by lung cDC2s, which are necessary for priming T helper cell responses to inhaled antigens.^44^, ^45^ NRP2 expression by cDCs has been reported to regulate DC–T cell interactions, but whether it affects T helper cell differentiation is unknown. Future studies will investigate if conditional deletion of NRP2 in DCs modulates T helper cell responses to inhaled antigens.

Recent studies indicate that impaired macrophage efferocytosis may contribute to the pathogenesis of neutrophilic asthma.^13^ Clearance of apoptotic immune and structural cells by airway macrophages is essential for the resolution of inflammation and maintenance of lung homeostasis.^29^ Efferocytosis of apoptotic cells prevents the release of damage‐associated molecular patterns that activate proinflammatory innate signaling pathways.^33^ Moreover, efferocytosis by macrophages inhibits proinflammatory cytokine production and induces the secretion of anti‐inflammatory mediators such as IL‐10 and TGF‐β.^33^, ^34^ Recently, NRP2 was shown to regulate macrophage efferocytosis by promoting the degradation of endocytosed apoptotic cells.^32^ Conditional deletion of NRP2 from tumor‐associated macrophages impaired the clearance of apoptotic tumor cells, which was associated with decreased expression of IL‐10 and TGF‐β and enhanced antitumor immune responses.^32^ Here, we show that NRP2 also regulates efferocytosis by AM. NRP2‐deficient AM exhibited impaired efferocytosis of apoptotic cells relative to wild‐type AM, which may contribute to the enhanced cellular airway inflammation in LysMcre/*Nrp2* ^*fl/fl*^ mice with neutrophilic asthma. In contrast to tumor‐associated macrophages, we did not detect changes in IL‐10 and TGF‐β production by NRP2‐deficient AM exposed to apoptotic cells, suggesting that NRP2 does not regulate the expression of these immunosuppressive cytokines in AM. NRP2 regulates endosomal maturation and endocytic transport of cell surface receptors,^46^ which could impact the uptake of apoptotic cells by AM. Polysialylation of NRP2 may also play a role in regulating efferocytosis by AM, as polysialylated NRP2 on peritoneal macrophages was reported to regulate phagocytosis of bacteria.^47^ Further studies are needed to determine the mechanisms by which NRP2 regulates AM efferocytosis and the role that impaired efferocytosis plays in neutrophilic asthma.

In summary, we have shown that NRP2 expression by lung macrophages is increased in a neutrophilic asthma model. Myeloid‐specific ablation of NRP2 leads to enhanced airway inflammation in mice with neutrophilic asthma, which was associated with impaired efferocytosis by AM. Overall, our findings suggest that NRP2 is an important negative regulator of neutrophilic asthma. Enhancement of NRP2 activity in the airways may be a novel treatment strategy for neutrophilic asthma and other TLR‐mediated inflammatory lung diseases.

## CONFLICT OF INTERESTS

The authors declare that there are no conflict of interests.

## AUTHOR CONTRIBUTIONS

Robert M. Immormino performed experiments and assisted with writing the manuscript. Corey M. Jania provided technical assistance with experiments. Stephen L. Tilley contributed to the interpretation of results and provided a critical review of the manuscript. Timothy P. Moran supervised the project, designed and performed experiments, interpreted results, and wrote the manuscript.

## Supporting information


**Supplemental Figure 1**. Gating strategy for multicolor flow cytometric analysis of lung leukocyte populations. Only single viable (Zombie Aqua^−^) cells were analyzed. *AM*, alveolar macrophages; *IM*, interstitial macrophages; *Inflamm Macs*, inflammatory monocyte‐derived macrophages; *cDCs*, conventional dendritic cells.

## Data Availability

The data that support the findings of this study are available from the corresponding author upon reasonable request.
