# Supplementary figures and images for: Neuropilin‐2 regulates airway inflammation in a neutrophilic asthma model
Source: Immun Inflamm Dis. 2021 Dec 3;10(3):e575. doi: 10.1002/iid3.575 (PMC8926497; doi:10.1002/iid3.575)

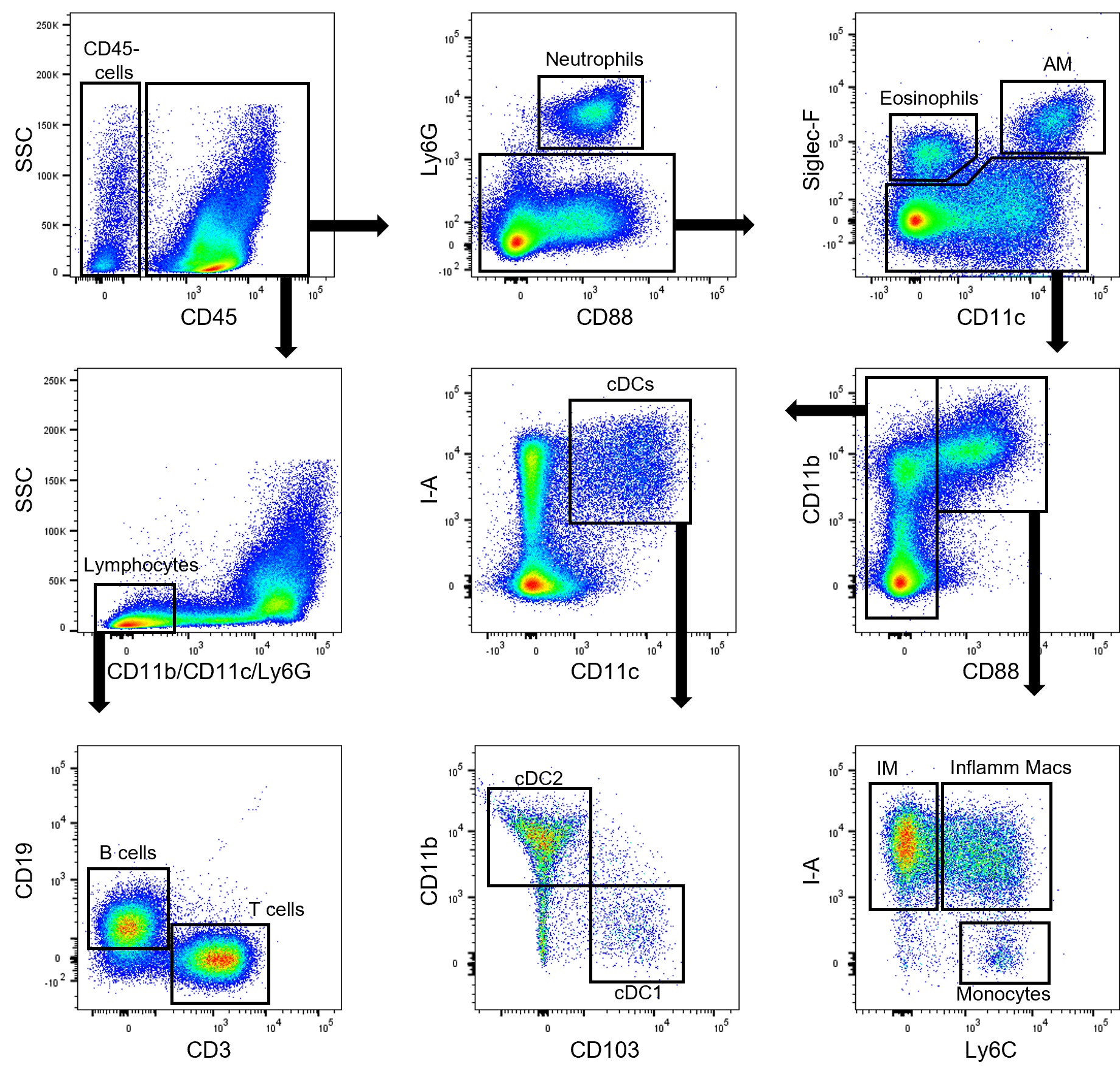

Supplement: Supplementary file 1 — Supplemental Figure 1. Gating strategy for multicolor flow cytometric analysis of lung leukocyte populations. Only single viable (Zombie Aqua−) cells were analyzed. AM, alveolar macrophages; IM, interstitial macrophages; Inflamm Macs, inflammatory monocyte‐derived macrophages; cDCs, conventional dendritic cells. [file IID3-10-e575-s001.tif]
